# Days at Home Among Dually Eligible Medicare Beneficiaries With Alzheimer Disease and Related Dementias

**DOI:** 10.1001/jamanetworkopen.2026.22670

**Published:** 2026-07-09

**Authors:** Yutong Zhang, Yang Yang, Johanna Thunell, Katherine E. M. Miller

**Affiliations:** 1 Department of Health Policy and Management, Johns Hopkins Bloomberg School of Public Health, Baltimore, Maryland; 2 Schaeffer Center for Health Policy and Economics, Sol Price School of Public Policy, University of Southern California, Los Angeles

## Abstract

This cross-sectional study examines US state-level variation in days at home among older dually eligible Medicare beneficiaries with Alzheimer disease and related dementias (ADRD).

## Introduction

More than 8 million US older adults (>65 years) are dually eligible for Medicare and Medicaid, representing one of the most vulnerable populations characterized by high health care use and spending.^1^ Alzheimer disease and related dementias (ADRD) are highly prevalent among older adults.^2^ Previous research indicates that dually eligible persons living with dementia (PLWD) have extensive health care use, including both acute care and home- and community-based services (HCBS) (eg, help with personal care).^3,4,5^

HCBS play a critical role in allowing PLWD to age in place, as aligned with preferences. HCBS access, financing, and delivery occurs primarily through Medicaid and varies substantially across states. Consequently, time spent at home among older dually eligible PLWD may also vary by state. Days at home is a key patient-centered outcome measure that can reflect periods of healthy state, stability, and independence and is correlated with increased quality of life.^6,7^ Yet less is known about geographic variation in time at home among vulnerable populations. Therefore, we examined variations in days at home among older dually eligible PLWD across states, including among HCBS users.

## Methods

The Johns Hopkins University Institutional Review Board approved this cross-sectional study. We used 2021 Medicare and Medicaid claims data to identify dually eligible beneficiaries who were fully enrolled in Medicaid for at least 1 month (eMethods in Supplement 1). We excluded enrollees in Centers for Medicare & Medicaid Services (CMS) Programs of All-Inclusive Care for the Elderly, Money Follows the Person, and Health Home and in managed care plans without observed claims; residents of Puerto Rico, the Virgin Islands, Mississippi, Nebraska, Michigan, and Ohio due to data quality concerns; and long-stay nursing home residents^8^ at the beginning of 2021. Informed consent was waived under 45 CFR §46.116(f)(3) criteria. We followed the STROBE reporting guideline.

We identified PLWD using the CMS Chronic Conditions Data Warehouse (CCW) flag and applied the CCW algorithm to encounter data to replicate an ADRD flag for Medicare Advantage beneficiaries. We defined our outcome—days at home—as the number of days per year a patient was alive without any emergency department, hospital, or nursing home use (ie, not in an inpatient, observation, skilled nursing facility, inpatient psychiatry, inpatient rehabilitation, or long-term hospital setting), as identified through the Medicare Provider Analysis and Review, Encounter, or Minimum Data Set 3.0 files.

We measured demographic characteristics (age, sex, race and ethnicity,^9^ US Census region, and urbanicity) and selected prevalent chronic conditions (hyperlipidemia, diabetes, stroke, depression, atrial fibrillation, and acute myocardial infarction). For all chronic conditions, we used CCW flags and replicated the algorithm in Encounter files. We identified HCBS use in Medicaid claims based on methodology described in a 2023 CMS brief.^10^ We described the sample characteristics, use of Medicaid-funded HCBS, and days at home. We then described state-level variation in days at home among the sample and conditional on HCBS user. Analyses were conducted from August 2025 to March 2026, using StataMP, version 18 (StataCorp LLC).

## Results

This study included 523 150 unique dually eligible PLWD (mean [SD] age, 82.0 [8.6] years; 40.8% aged >85 years; 68.1% female and 31.9% male) (Table). One-tenth (9.2%) of PLWD resided in rural areas, 61.0% used any HCBS, and 42.5% had depression. Older dually eligible PLWD had a mean (SD) 238.1 (144.8) days at home in 2021. Among HCBS users, PLWD had a mean (SD) 275.0 (126.6) days at home.

**Table.** Demographic Characteristics of Older Dually Eligible Medicare Beneficiaries With Alzheimer Disease and Related Dementias, 2021^a^

Characteristic	Value (N = 523 150)
Age, y	
Mean (SD)^b^	82.0 (8.6)
>85	213 522 (40.8)
Sex	
Female	356 198 (68.1)
Male	166 952 (31.9)
Race and ethnicity^c^	
American Indian or Alaska Native	3725 (0.7)
Asian or Pacific Islander	41 440 (7.9)
Hispanic	96 723 (18.5)
Non-Hispanic Black	84 013 (16.1)
Non-Hispanic White	285 814 (54.6)
Other or unknown	11 435 (2.2)
US Census region	
Midwest	72 834 (13.9)
Northeast	135 394 (25.9)
South	178 639 (34.1)
West	136 283 (26.1)
Area of residence	
Metropolitan	428 212 (81.9)
Micropolitan	46 885 (9.0)
Rural	48 015 (9.2)
Missing	38 (<0.01)
Chronic condition^d^	
Hyperlipidemia	367 316 (70.2)
Diabetes	248 327 (47.5)
Stroke	86 677 (16.6)
Depression	222 592 (42.5)
Atrial fibrillation	107 896 (20.6)
Acute myocardial infarction	25 232 (4.8)
HCBS user	319 212 (61.0)
Days at home	
Mean (SD)	238.1 (144.8)
Mean (SD) conditional on using HCBS	275.0 (126.6)

Abbreviation: HCBS, home- and community-based services.

^a^ Unless indicated otherwise, values are No. (%) of beneficiaries.

^b^ Age at the end of the reference year.

^c^ Based on Research Triangle Institute (RTI) race and ethnicity variables. The RTI race variable improves accuracy of race and ethnicity classification, particularly for Asian, Hispanic, and Pacific Islander individuals. Other race or ethnicity captures beneficiaries who are non-Hispanic and do not fall into the primary racial and ethnic groups defined by the measure.^9^

^d^ Identified by both Centers for Medicare & Medicaid Services Chronic Condition Warehouse and Medicare Advantage data.

Across states, there was substantial variation in the mean (SD) number of days at home, ranging from 159.3 (144.7) days in Kentucky to 311.7 (102.4) days in Alaska in 2021 (Figure). Older dually eligible PLWD residing in Alabama, Rhode Island, and Missouri had fewer than 195 days at home (mean [SD], 186.1 [141.1], 190.8 [146.4], and 192.5 [149.8] days, respectively); those residing in Washington, California, and Oregon had more than 270 days at home (275.4 [127.3], 276.3 [132.1], and 282.5 [121.4] days, respectively). This variation across states persisted when conditioned on any HCBS use. Across states, HCBS users had more days at home than the overall population; however, the magnitude of this difference varied across states.

**Figure.**
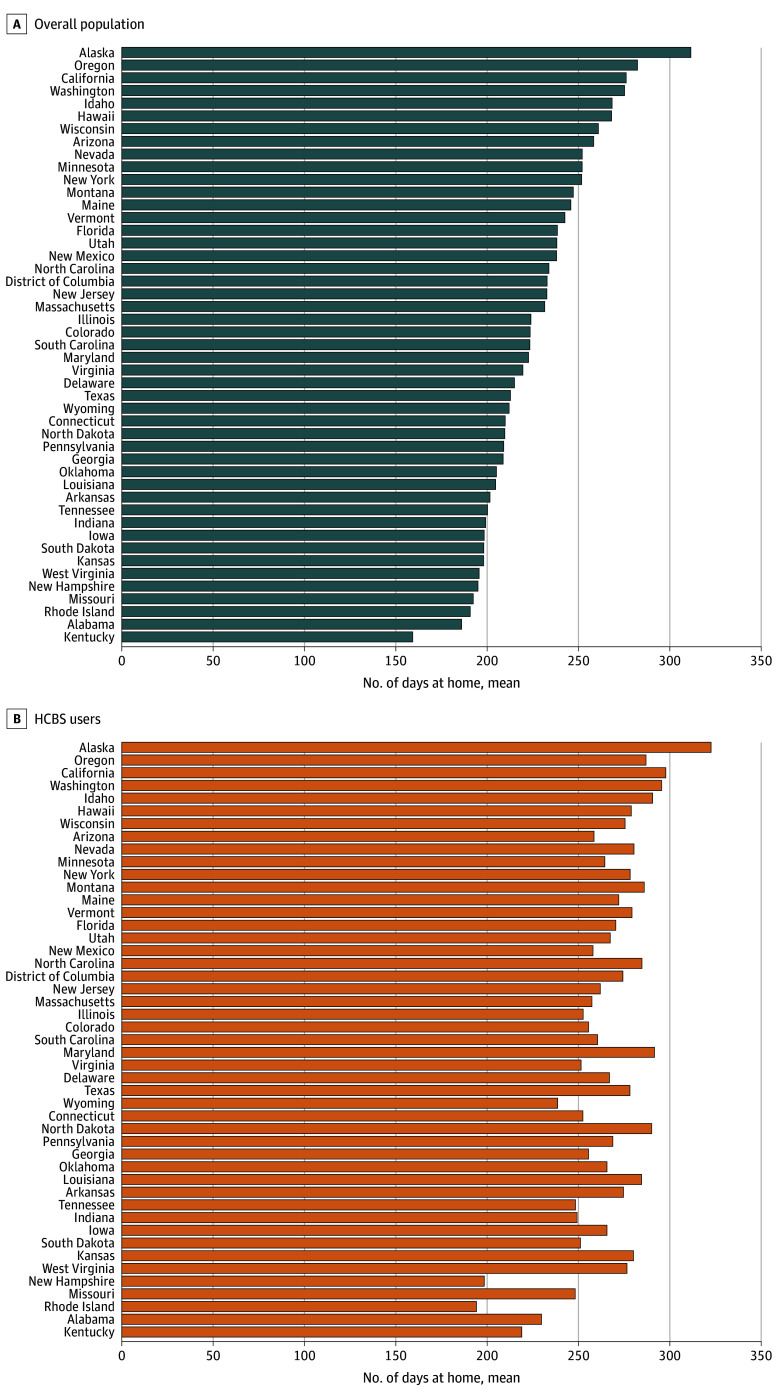
Bar Graphs of Average Days at Home by State Among Older Dual-Eligible Beneficiaries With Alzheimer Disease and Related Dementias, Overall Population and Medicaid Home- and Community-Based Services (HCBS) Users Observations from Puerto Rico, the Virgin Islands, Mississippi, Nebraska, Michigan, and Ohio were excluded due to data quality concerns regarding Medicaid claims for HCBS. Days at home was defined as the number of days per year a patient was alive without any emergency department, hospital, or nursing home use (ie, the patient is not in an inpatient, observation, skilled nursing facility, inpatient psychiatry, inpatient rehabilitation, or long-term hospital setting), as identified through the Medicare Provider Analysis and Review, Encounter, or Minimum Data Set 3.0 files. Individuals who were long-stay nursing home residents during the first 4 months of the year, defined as residing in nursing homes for at least 100 cumulative days with no more than 30 days spent outside the facility during that period, were also excluded.^8^

## Discussion

In this nationwide cross-sectional study, older dually eligible PLWD spent a mean 238 days at home. The substantial variation observed across states may reflect differences in local health service supply, state Medicaid HCBS policies and program design, family caregiving availability, and patient preferences.

Study limitations include reduced generalizability owing to exclusion of some states with Medicaid managed care programs, potential bias from differential attrition across states due to mortality, and limited information on beneficiaries’ health and functional status. While we are unable to disentangle whether variation stemmed from patient preferences or barriers to accessing care, our findings highlight the imperative to further identify factors that may increase days at home for vulnerable populations, including older dually eligible PLWD.

## Notes

Supplement 1.
eMethods

eReferences

Supplement 2.
Data Sharing Statement
