# Supplementary material for: Days at Home Among Dually Eligible Medicare Beneficiaries With Alzheimer Disease and Related Dementias
Source: JAMA Netw Open. 2026 Jul 9;9(7):e2622670. doi: 10.1001/jamanetworkopen.2026.22670 (PMC13352127; doi:10.1001/jamanetworkopen.2026.22670)
Supplement: Supplement 1. — eMethods eReferences [file jamanetwopen-e2622670-s001.pdf]

Supplementary Online Content

Zhang Y, Yang Y, Thunell J, Miller KEM. Days at home among dually eligible Medicare beneficiaries with Alzheimer disease and related dementias. *JAMA Netw Open*. 2026;9(7):e2622670. doi:10.1001/jamanetworkopen.2026.22670

eMethods

eReferences

This supplementary material has been provided by the authors to give readers additional information about their work.

© 2026 Zhang Y et al. *JAMA Network Open*.

eMethods

Sample Construction

Using the Master Beneficiary Summary File, we identified dually eligible beneficiaries who were 65+ and fully enrolled in Medicaid for ≥1 month. We then excluded enrollees in Programs of All-Inclusive Care for the Elderly (PACE), Money Follows the Person, Health Home, and managed care plans without observed claims. We excluded PACE and Health Home enrollees because of the limited claims data to identify types of HCBS use, as detailed in the CMS 2023 Issue Brief;[1] We then further excluded residents of Puerto Rico, Virgin Islands, Mississippi, Nebraska, Michigan, and Ohio due to data quality concerns based on individual analyses and as informed by the DQ Atlas. Finally, we excluded individuals who are long-stay nursing home residents at the beginning of 2021. We identified long-stay nursing home residents during the first four months of the year, defined as residing in nursing homes for at least 100 cumulative days with no more than 30 days spent outside the facility during that period.[2] Therefore, if someone met criteria for a long-stay as of April 30, 2021, they were excluded from analyses.

We did reproduce all findings retaining these initial long-stay nursing home residents and findings were robust and are available upon request.

© 2026 Zhang Y et al. *JAMA Network Open*.

eReferences

1. Stepanczuk C, Murray C, Carpenter A, Larsen A, Wysocki A. *Methodology for Identifying Medicaid Long-Term Services and Supports Expenditures and Users, 2023*. Mathematica; 2025. Accessed March 24, 2026. https://www.medicaid.gov/medicaid/long-term-services-supports/downloads/ltss-users-ident-method-2023.pdf
2. Centers for Medicare & Medicaid Services. *MDS 3.0 Quality Measures User's Manual (Version 18.0)*. 2026. Accessed March 24, 2026. https://cdn.ymaws.com/www.leadingagesoutheast.org/resource/resmgr/covid-19/2025/12/mds-3.0-qm-users-manual-v18..pdf

© 2026 Zhang Y et al. *JAMA Network Open*.
