# Supplementary material for: Days at Home Among Dually Eligible Medicare Beneficiaries With Alzheimer Disease and Related Dementias
Source: JAMA Netw Open. 2026 Jul 9;9(7):e2622670. doi: 10.1001/jamanetworkopen.2026.22670 (PMC13352127; doi:10.1001/jamanetworkopen.2026.22670)
Supplement: Supplement 2. — Data Sharing Statement [file jamanetwopen-e2622670-s002.pdf]

Data Sharing Statement

Zhang. Days at Home Among Dually Eligible Medicare Beneficiaries With Alzheimer Disease and Related Dementias. *JAMA Netw Open*. Published July 09, 2026. doi:10.1001/jamanetworkopen.2026.22670

Data
Data available: No
